# Texture analysis of cartilage T_2 _maps: individuals with risk factors for OA have higher and more heterogeneous knee cartilage MR T_2 _compared to normal controls - data from the osteoarthritis initiative

**DOI:** 10.1186/ar3469

**Published:** 2011-09-20

**Authors:** Gabby B Joseph, Thomas Baum, Julio Carballido-Gamio, Lorenzo Nardo, Warapat Virayavanich, Hamza Alizai, John A Lynch, Charles E McCulloch, Sharmila Majumdar, Thomas M Link

**Affiliations:** 1 Department of Radiology and Biomedical Imaging, University of California San Francisco, 1700 4th Street, Suite 203, San Francisco, CA 94107, USA; 2 Department of Epidemiology and Biostatistics, University of California San Francisco, 185 Berry Street, Lobby 5, Suite 5700, San Francisco, CA 94107, USA

## Abstract

**Introduction:**

The goals of this study were (i) to compare the prevalence of focal knee abnormalities, the mean cartilage T_2 _relaxation time, and the spatial distribution of cartilage magnetic resonance (MR) T_2 _relaxation times between subjects with and without risk factors for Osteoarthritis (OA), (ii) to determine the relationship between MR cartilage T_2 _parameters, age and cartilage morphology as determined with whole-organ magnetic resonance imaging scores (WORMS) and (iii) to assess the reproducibility of WORMS scoring and T_2 _relaxation time measurements including the mean and grey level co-occurrence matrix (GLCM) texture parameters.

**Methods:**

Subjects with risk factors for OA (n = 92) and healthy controls (n = 53) were randomly selected from the Osteoarthritis Initiative (OAI) incidence and control cohorts, respectively. The specific inclusion criteria for this study were (1) age range 45-55 years, (2) body mass index (BMI) of 19-27 kg/m^2^, (3) Western Ontario and McMaster University (WOMAC) pain score of zero and (4) Kellgren Lawrence (KL) score of zero at baseline. 3.0 Tesla MR images of the right knee were analyzed using morphological gradings of cartilage, bone marrow and menisci (WORMS) as well as compartment specific cartilage T_2 _mean and heterogeneity. Regression models adjusted for age, gender, and BMI were used to determine the difference in cartilage parameters between groups.

**Results:**

While there was no significant difference in the prevalence of knee abnormalities (cartilage lesions, bone marrow lesions, meniscus lesions) between controls and subjects at risk for OA, T_2 _parameters (mean T_2_, GLCM contrast, and GLCM variance) were significantly elevated in those at risk for OA. Additionally, a positive significant association between cartilage WORMS score and cartilage T_2 _parameters was evident.

**Conclusions:**

Overall, this study demonstrated that subjects at risk for OA have both higher and more heterogeneous cartilage T_2 _values than controls, and that T_2 _parameters are associated with morphologic degeneration.

## Introduction

Osteoarthritis (OA) is a degenerative joint disease that affects more than 27 million people in the US alone [1]. OA is characterized by biochemical and morphologic degradation of joint tissues (in particular, the articular hyaline cartilage). The process of cartilage loss is manifested by biochemical degeneration (proteoglycan loss, increased water content, collagen degradation, and chondrocyte response to tissue damage) as well as morphologic degeneration such as fibrillation and cartilage thinning [2,3]. Biochemical alterations to the articular cartilage often occur prior to morphologic degeneration [4]; thus, evaluating the biochemical composition of cartilage may be valuable for the early detection of OA.

Magnetic resonance (MR) T_2 _relaxation time is sensitive to biochemical changes that occur during cartilage degeneration, including alterations in hydration, collagen content, and tissue anisotropy [5]. Mean cartilage T_2 _has been used to distinguish subjects with early OA from healthy subjects [6]. Recent studies have suggested that, in addition to mean T_2_, the spatial distribution of cartilage T_2 _values may be important when examining the pathogenesis of OA [7-9]. Early degenerative changes of the cartilage matrix due to disease or injury are reflected by the spatial distribution of T_2 _values and can be quantified by grey level co-occurrence matrix (GLCM) texture analysis [10]. GLCM entropy of cartilage T_2 _has been found to be elevated in patients with OA as compared with controls [7,9], demonstrating that not only mean T_2 _[6] but also the spatial distribution of T_2 _values is affected by disease.

The Osteoarthritis Initiative (OAI) is a multi-center longitudinal study aimed at assessing biomarkers in OA, including those derived from MR imaging (MRI). The OAI is a cross-sectional and longitudinal dataset that includes both MRI and radiographic images of subjects, scanned annually over 4 years. MR images that can be used to assess joint morphology and cartilage T_2 _are available. This database provides a means to longitudinally evaluate MRI biomarkers, including T_2 _relaxation time in the development and progression of OA, thus providing a wealth of information on OA development and progression.

While many previous studies have evaluated subjects with symptomatic and radiographic OA [11-13], the present study evaluates subjects at risk for developing OA (but without radiographic knee degeneration or pain within the week before MRI) as well as normal controls. This patient cohort is unique, facilitating the assessment of early biochemical changes in OA which occur prior to morphologic degeneration detected by radiography. Since early morphologic degeneration in the joint may not be detected by radiography [14,15], this study uses MRI to assess cartilage and meniscus morphology. The MR whole-organ magnetic resonance imaging scores (WORMS) [16] are employed for focal knee evaluation, and MR T_2 _relaxation time is used for the assessment of cartilage biochemical composition. The goals of this study were (a) to compare the prevalence of focal knee abnormalities, the mean cartilage T_2 _relaxation time, and the spatial distribution of cartilage MR T_2 _relaxation times between subjects with risk factors for OA and those without them; (b) to determine the relationship between MR cartilage T_2 _parameters, age, and cartilage morphology as determined by WORMS; and (c) to assess the reproducibility of WORMS scoring and T_2 _relaxation time measurements, including the mean and GLCM texture parameters.

## Materials and methods

### Subjects

A subset of subjects from the incidence (n = 92) and control (n = 53) cohorts of the OAI [17] was selected on the basis of the inclusion criteria of this study. The incidence cohort did not have symptomatic knee OA - criteria were no 'frequent knee symptoms in the past 12 months, defined as "pain, aching, or stiffness in or around the knee on most days" for at least 1 month during the past 12 months, and no radiographic tibiofemoral knee OA, defined as definite tibiofemoral osteophytes (Osteoarthritis Research Society International atlas grades 1 to 3, equivalent to Kellgren-Lawrence (KL) grade of at least 2 on fixed flexion radiographs in either knee at baseline)' [17] - but did have risk factors for OA, including being overweight (defined using gender- and age-specific cut-points for weight: males of greater than 92.9 kg and females of greater than 77.1 kg from the age of 45 to 69 years) or having knee injury (defined as a history of knee injury causing difficulty walking for at least 1 week), knee surgery (defined as a history of knee surgery, including meniscal and ligamentous repairs and unilateral total knee replacement for OA), family history of total knee replacement (defined as a total knee replacement for OA in a biological parent or sibling), or Heberden nodes (defined as self-report of bony enlargement of 1+ distal interphalangeal joint in both hands) [17]. Subjects from the control cohort had no knee symptoms or risk factors for OA. The exclusion criteria for the study included rheumatoid arthritis, bilateral total knee joint replacement, and a positive pregnancy test. The specific inclusion criteria for this study were (a) age range of 45 to 55 years, (b) body mass index (BMI) of 19 to 27 kg/m^2^, (c) Western Ontario and McMaster Universities Osteoarthritis Index (WOMAC) pain score of 0, and (d) KL score of 0 at baseline. These parameters were chosen in order to examine a middle-aged, non-obese, and asymptomatic population without radiographic evidence of OA. The following OAI datasets were assessed in this study: baseline clinical dataset 0.2.2 and baseline imaging datasets 0.E.1 and 0.C.2. The institutional review boards at all units participating in the OAI, including the clinical centers and the OAI Coordinating Center at University of California San Francisco, have reviewed and approved the protocol and consent forms for the OAI study. All OAI study participants signed informed consent forms for participation in the study.

### Knee radiographs

Bilateral standing posterior-anterior fixed flexion knee radiographs were acquired at baseline. Knees were positioned in a plexiglass frame (SynaFlexer; CCBR-Synarc, Newark, CA, USA) with 20° to 30° flexion and 10° internal rotation of the feet. In an additional reading performed for the present study, knee radiographs were graded by two radiologists (LN and WV) in consensus by using the KL scoring system [18]. The KL score included only the tibiofemoral joint and not the patellofemoral joint since the OAI used the posterior-anterior 'fixed flexion' knee radiograph protocol, which is a primary protocol for tibiofemoral joint radiography.

### Magnetic resonance imaging

MR images were obtained with four identical 3.0 Tesla scanners (Siemens Magnetom Trio, Erlangen, Germany) and quadrature transmit-receive coils (USA Instruments, Aurora, OH, USA) in Columbus, OH; Baltimore, MD; Pittsburgh, PA; and Pawtucket, RI. The following sequences were acquired and used for image analysis: sagittal two-dimensional (2D) intermediate-weighted (IW) fast spin-echo (FSE) sequence (resolution = 0.357 × 0.511 × 3.0 mm) and a coronal 2D IW FSE sequence (resolution = 0.365 × 0.456 × 3.0 mm). A sagittal 2D multi-slice multi-echo (MSME) sequence (TE_1_-TE_7 _= 10, 20, 30, 40, 50, 60, 70 ms, resolution = 0.313 × 0.446 × 3.0 mm, and 0.5 mm gap) was used for T_2 _measurements [19].

### Image analysis

All images were analyzed with a Sun Workstation (Sun Microsystems, now part of Oracle Corporation, Redwood Shores, CA, USA). Knee articular cartilage was segmented manually in five compartments (patella, medial femur, medial tibia, lateral femur, and lateral tibia) as previously reported [20,21]. An IDL (Interactive Data Language, Research Systems, Boulder, CO, USA) software routine was implemented to manually segment the cartilage from the T_2 _maps by one operator (HA). Segmentation was performed on a slice-by-slice basis (spanning all slices), and each region of interest encompassed the entirety of the cartilage tissue. To exclude potential chemical shift artifacts or fluid from the region of interest, the user simultaneously examined the T_2 _map and the first echo of the MSME sequence (in neighboring image panels) with synchronized cursor, slice number, and zoom.

T_2 _maps were computed on the basis of Equation 1 from the MSME images on a pixel-by-pixel basis by using six echoes (TE = 20 to 70 ms) and three parameter fittings accounting for noise [22,23].

(1) $$S{\left(TE\right)}^{2}={S_{0}}^{2}e^{-\frac{2*TE}{T_{2}}}+B^{2}$$

In Equation 1, S is the signal intensity at a given echo time (TE), S_0 _is the signal intensity at TE = 0 ms, and B is the estimated noise at a given TE. To reduce potential errors resulting from stimulated echoes in a multi-echo Carr-Purcell-Meiboom-Gill sequence [24,25], the first echo (TE = 10 ms) was not included in the T_2 _fitting procedure. A noise-corrected algorithm was implemented based on results from a recent study demonstrating increased accuracy and precision of T_2 _relaxation time when using a noise-corrected algorithm as compared with the traditional uncorrected exponential fit [22,23]. T_2 _quantification was performed with an in-house program created with Matlab (MathWorks, Natick, MA, USA).

### Texture analysis

Texture analysis was performed on a slice-by-slice basis on the cartilage T_2 _maps. This method is based on the GLCM as described by Haralick and colleagues [10]. The GLCM determines the frequency that neighboring grey-level values occur in an image. GLCM texture parameters, including contrast, variance, and entropy, were calculated in each cartilage region. The equations for contrast, variance, and entropy are shown below (Equations 2-4), respectively.

(2) $$Entropy=\overset{N}{\underset{i=1}{\sum }}\overset{N}{\underset{j=1}{\sum }}P\left(i,j\right)\left(-\ln P\left(i,j\right)\right)$$

(3) $$Variance=\overset{N-1}{\underset{i,j=0}{\sum }}P_{i,j}{\left(i-\mu_{i,j}\right)}^{2}$$

Where

$$\mu_{i,j}=\overset{N^{\prime \prime }1}{\underset{i,j=0}{\#}}i\left(P_{i,j}\right)$$

(4) $$Contrast=\overset{N}{\underset{i=1}{\sum }}\overset{N}{\underset{j=1}{\sum }}P\left(i,j\right){\left(i-j\right)}^{2}$$

P represents the probability of the co-occurrence of pixel values i and j in an image. N represents the total number of pixel value co-occurrences in the image. A pixel offset of one pixel was chosen based on the fact that approximately three to four pixels span the cartilage thickness. Analysis was performed by averaging the GLCM parameters across four orientations: 0° (corresponding to the anterior-posterior axis), 45°, 90° (corresponding to the superior-inferior axis), and 135°.

### WORMS scoring

MR images of the right knee were reviewed on picture archiving communication system workstations (Agfa, Ridgefield Park, NJ, USA). A board-certified radiologist (WV) with 7 years of experience and a fourth-year radiology resident (LN) with 3 years of experience read the images independently and graded meniscus, cartilage, and bone marrow lesions. Cartilage and bone marrow lesions were assessed in five compartments (patella, medial femur, medial tibia, lateral femur, and lateral tibia) by using a modified semi-quantitative WORMS [16,26,27], and the highest grade of lesion was recorded for each region. In case of disagreement, a consensus reading was performed with a musculoskeletal radiologist with 22 years of experience (TML). For calibration purposes, the first 20 cases were read simultaneously by the three readers in consensus. Compared with the original WORMS grading system, only six compartments were analyzed as relatively mild lesions were expected. This could have potentially affected the number of grade 4 or grade 6 cartilage lesions as well as grade 3 bone marrow lesions, all of which, however, are rare. Cartilage signal and morphology were scored with an 8-point scale: 0 = normal thickness and signal, 1 = normal thickness but increased signal on T_2_-weighted images, 2.0 = partial-thickness focal defect of less than 1 cm in greatest width, 2.5 = full-thickness focal defect of less than 1 cm in greatest width, 3 = multiple areas of partial-thickness (grade 2.0) defects intermixed with areas of normal thickness or a grade 2.0 defect of wider than 1 cm but less than 75% of the region, 4 = diffuse (at least 75% of the region) partial-thickness loss, 5 = multiple areas of full-thickness loss (grade 2.5) or a grade 2.5 lesion of wider than 1 cm but less than 75% of the region, and 6 = diffuse (at least 75% of the region) full-thickness loss. Meniscal morphology was assessed in six regions by using a modified WORMS: the medial and lateral sides of the anterior, body, and posterior regions; an additional grade was added to the meniscal classification 'intrasubstance degeneration' to better assess early degenerative disease. The grading scale ranged from 1 to 4: 0 = normal, 1 = intrasubstance abnormalities, 2 = non-displaced tear, 3 = displaced or complex tear, and 4 = complete destruction. Subarticular bone marrow abnormalities were defined as poorly marginated areas of increased signal intensity in the normal subchondral and epiphyseal bone marrow on T_2_-weighted FSE fast-suppressed MR images. A 4-point grading scale was employed to assess the size of the bone marrow abnormalities: 0 = none, 1 = minimal (less than 25% of region), 2 = moderate (25% to 50% of region), and 3 = severe (greater than 50% of region) [20].

### Reproducibility

The reproducibility of WORMS scoring for meniscus, cartilage, and bone marrow tissues was investigated in 15 subjects and read out twice by two radiologists independently. An intraclass correlation coefficient (ICC) was calculated to determine the intra- and inter-reader reproducibility errors [28]. The ICC is mathematically equal to the weighted kappa using quadratic weights [29,30]. The reproducibility of mean T_2 _and texture analysis was determined by segmenting the cartilage in five subjects, three times by one operator (HA). The reproducibility error was calculated as the root mean square (RMS) coefficient of variation (CV) of the repeated measurements as described by Glüer and colleagues [31].

### Statistical analysis

Statistical analysis was performed with STATA 11 software (StataCorp LP, College Station, TX, USA). Descriptive statistics (i.e. mean age, gender, and BMI) were calculated for each group, and differences between groups were assessed by using regression models and a Pearson chi-square test. The association between age and mean T_2 _was assessed by using regression models and partial correlations adjusting for group, gender, and BMI.

The primary compartmental predictors of this study were the medial femur, the medial tibia, and the average of all compartments. The medial femur and medial tibia were chosen based on the following rationale: the medial side of the knee is a concentrated region of weight-bearing [32], the medial side of the knee has a higher incidence of OA than the lateral side [33], and meniscal and cartilage lesions are more prevalent on the medial side of the joint [33]. The remaining compartments, including the lateral femur, lateral tibia, and patella, were examined in an exploratory manner. Additionally, three GLCM texture parameters were analyzed (GLCM contrast, GLCM variance, and GLCM entropy) and were regarded as representative parameters from each of the three texture groups (contrast, statistics, and order, respectively). These texture parameters were selected based on of results from previous studies demonstrating their elevation in subjects with OA [7-9].

Two separate analyses were performed to assess the prevalence of morphologic knee abnormalities in each group: the first analysis defined the prevalence of cartilage (and meniscus) lesions as present for any compartment that had WORMS of greater than 0. The second analysis defined prevalence as present for any compartment that had WORMS of at least 2. The rationale for these chosen cutoff points was to assess subjects with any features of cartilage degeneration (WORMS of greater than 0) and subjects with mild degeneration (WORMS of at least 2). The prevalence of subjects with severe degeneration (WORMS of greater than 4) [34] was scarce (5 subjects overall); thus, this study did not focus on these subjects. The differences in the prevalence of morphologic knee abnormalities between groups were assessed by using logistic regression models (independent variable: group; dependent variable: WORMS prevalence). The prevalence of bone marrow lesions was defined as a BML score greater than 0, and a logistic regression model (described above) was performed to assess the differences in the prevalence of bone marrow lesions between groups.

The differences in T_2 _parameters between control group (CG) and incidence group (IG) were assessed by using regression models (independent variables: group; dependent variable: T_2 _parameters). To compare the differences in T_2 _parameters between groups, the following equation was implemented: (T_2__parameter_IG _- T_2__parameter_CG_)/(the average standard deviation (SD) of both groups).

The relationship between the prevalence of morphologic abnormalities and cartilage T_2 _was investigated by using regression models and partial correlations (independent variables: T_2 _parameter and group; dependent variable: 'WORMS max score'). The WORMS max score is defined as the maximum of the WORMS in all compartments per patient. All models were adjusted for age, gender, and BMI.

## Results

The control (n = 53) and incidence (n = 92) groups had no significant differences (*P *> 0.05) in age or BMI (age_control _= 50.30 ± 3.03 years, age_incidence _= 50.65 ± 2.89 years, *P *= 0.49; BMI_control _= 23.90 ± 2.23 kg/m^2^, BMI_incidence _= 23.78 ± 2.25 kg/m^2^, *P *= 0.78). The incidence group consisted of 50 (54.34%) females, whereas the control group consisted of 36 (67.92%) (*P *> 0.05) (Table 1). The incidence group had the following distribution of risk factors: 44 had a previous injury, 19 had previous knee surgery, 19 had a family history of knee replacement, and 17 had Heberden nodes.

**Table 1:** Subject characteristics

Characteristic	Incidence cohort	Control cohort	*P *value
Number of subjects	92	53	
Age in years, mean ± SD	50.65 ± 2.89	50.30 ± 3.03	0.49^a^
Body mass index in kg/m^2^, mean ± SD	23.78 ± 2.25	23.90 ± 2.23	0.78^a^
Number of females	50	36	0.10^b^
WOMAC pain score	0	0	
Kellgren-Lawrence score	0	0	

^a^Regression model; ^b^Pearson chi-square test. SD, standard deviation; WOMAC, Western Ontario and McMaster Universities Osteoarthritis Index.

The reproducibility results for the WORMS grading are listed in Table 2. The intra-observer reproducibility in all tissues (meniscus, cartilage, and bone marrow) was at least 96%, whereas the inter-observer reproducibility was at least 97%. The reproducibility results for the mean cartilage T_2 _and the GLCM texture analysis are listed in Table 3. The mean T_2 _values had RMS CVs ranging from 0.85% in the lateral femur to 2% in the medial tibia. GLCM entropy exhibited the lowest CVs (<1%), whereas GLCM contrast had CVs of less than 5% in all compartments except for the lateral tibia (8.67%) and medial tibia (11.41%). The CVs for GLCM variance were less than 5%, except for the medial tibia, which had a CV of 7.91%.

**Table 2:** Interclass correlation coefficient [28] of the whole-organ magnetic resonance imaging scores (WORMS) in the meniscus, cartilage, and bone marrow lesions

Tissue	Reader 1	Reader 2	ICC
Meniscus	0.98	0.96	0.97
Cartilage	0.98	0.96	0.98
Bone marrow lesions	0.98	0.97	0.98

The reproducibility of WORMS scores was investigated in 15 subjects and read out twice by two readers independently. ICC, interclass correlation coefficient.

**Table 3:** Reproducibility (coefficient of variation) [31] of T_2 _measurements in five subjects segmented three times each by one operator

	Medial		Lateral		
Parameter	Femur	Tibia	Femur	Tibia	Patella
Mean T_2_	0.99	2.08	0.85	1.51	0.86
Contrast	4.60	11.41	3.61	8.67	4.07
Entropy	0.47	0.75	0.05	0.03	0.55
Variance	2.40	7.91	2.12	4.31	3.34

The texture parameters analyzed were at 0 degrees and at 1 pixel offset.

A significant association (*r *= 0.19, *P *= 0.04) was evident between subject age and mean T_2 _(adjusting for group, gender, and BMI) in all compartments combined for both the incidence and control groups. The GLCM texture parameters and WORMS max scores were not significantly related to age (*P *> 0.05) in both groups.

The prevalence of focal knee abnormalities (cartilage lesions, bone marrow lesions, and meniscus lesions) was not significantly (*P *> 0.05) different between the incidence and control groups (Tables 4, 5, 6). No significant differences between groups were observed when evaluating the overall prevalence of knee abnormalities (Table 4) or the prevalence of knee abnormalities by compartment (Tables 5 and 6). The patella had the highest prevalence of cartilage defects (WORMS of greater than 0: 54.5% in the incidence cohort and 57.7% in the control cohort), followed by the lateral tibia (WORMS of greater than 0: 19.3% in the incidence cohort and 22.2% in the control cohort). Also, mensical tears were most abundant in the medial posterior compartment (WORMS of greater than 0: 43.1% in the incidence group and 33.3% in the control group), followed by the medial body (WORMS of greater than 0: 12.5% in the incidence group and 20.0% in the control group).

**Table 4:** Prevalence of focal knee abnormalities in the incidence and control groups

Lesion	**Control group**^**a **^**(n = 53)**	**Incidence group**^**a **^**(n = 92)**	**Odds ratio**^**b**^	95% confidence interval	
Cartilage					
WORMS >0	33 (73.3%)	59 (67.0%)	0.83	0.36	1.88
WORMS ≥2	19 (42.2%)	44 (50.0%)	1.42	0.66	3.05
Bone marrow lesions	19 (42.2%)	29 (32.9%)	0.64	0.29	1.40
Meniscus					
WORMS >0	18 (40.0%)	42 (47.7%)	1.52	0.71	3.30
WORMS ≥2	9 (15.5%)	22 (25.0%)	2.16	0.73	6.35

^a^Eight subjects from the control group and four subjects from the incidence group did not have whole-organ magnetic resonance imaging score (WORMS) readings available. ^b^All *P *values were greater than 0.05 using a logistic regression both when unadjusted and when adjusted for age, body mass index, and gender.

**Table 5:** Prevalence of cartilage abnormalities (cartilage WORMS >0 and WORMS ≥2) in the incidence and control groups by compartment

Prevalence of WORMS >0					
	Medial		Lateral		
Group^a, b^	Femur	Tibia	Femur	Tibia	Patella
Incidence group (n = 92)	11 (12.5%)	4 (4.5%)	8 (9.0%)	17 (19.3%)	48 (54.5%)
Control group (n = 53)	5 (11.1%)	1 (2.2%)	2 (4.4%)	10 (22.2%)	26 (57.7%)
Prevalence of WORMS ≥2					
	Medial		Lateral		
Group^a, b^	Femur	Tibia	Femur	Tibia	Patella
Incidence group (n = 92)	10 (11.3%)	3 (3.4%)	6 (6.8%)	10 (11.3%)	25 (28.4%)
Control group (n = 53)	3 (6.6%)	0 (0.0)%	1 (2.2%)	4 (8.8%)	11 (24.4%)

^a^All *P *values were greater than 0.05 using a logistic regression both when unadjusted and when adjusted for age, body mass index, and gender. ^b^Eight subjects from the control group and four subjects from the incidence group did not have whole-organ magnetic resonance imaging score (WORMS) readings available. Various subjects had lesions in multiple compartments.

**Table 6:** Prevalence of meniscus abnormalities (meniscus WORMS >0 and WORMS ≥2) in the incidence and control groups by compartment

Prevalence of WORMS >0						
	Medial			Lateral		
Group^a, b^	Anterior	Body	Posterior	Anterior	Body	Posterior
Incidence group (n = 92)	1 (1.1%)	11 (12.5%)	38 (43.1%)	6 (6.8%)	4 (4.5%)	10 (11.3%)
Control group (n = 53)	0 (0.0%)	9 (20.0%)	15 (33.3%)	1 (2.2%)	0 (0.0%)	5 (11.1%)
Prevalence of WORMS ≥2						
	Medial			Lateral		
Group^a, b^	Anterior	Body	Posterior	Anterior	Body	Posterior
Incidence group (n = 92)	0 (0.0%)	7 (8.3%)	18 (26.4%)	3 (3.5%)	1 (1.1%)	0 (0.0%)
Control group (n = 53)	0 (0.0%)	5 (12.2%)	5 (14.2%)	1 (2.2%)	0 (0.0%)	1 (2.4%)

^a^All *P *values were greater than 0.05 using a logistic regression both when unadjusted and when adjusted for age, body mass index, and gender. ^b^Eight subjects from the control group and four subjects from the incidence group did not have whole-organ magnetic resonance imaging score (WORMS) readings available. Various subjects had lesions in multiple compartments.

**Figure 1:**
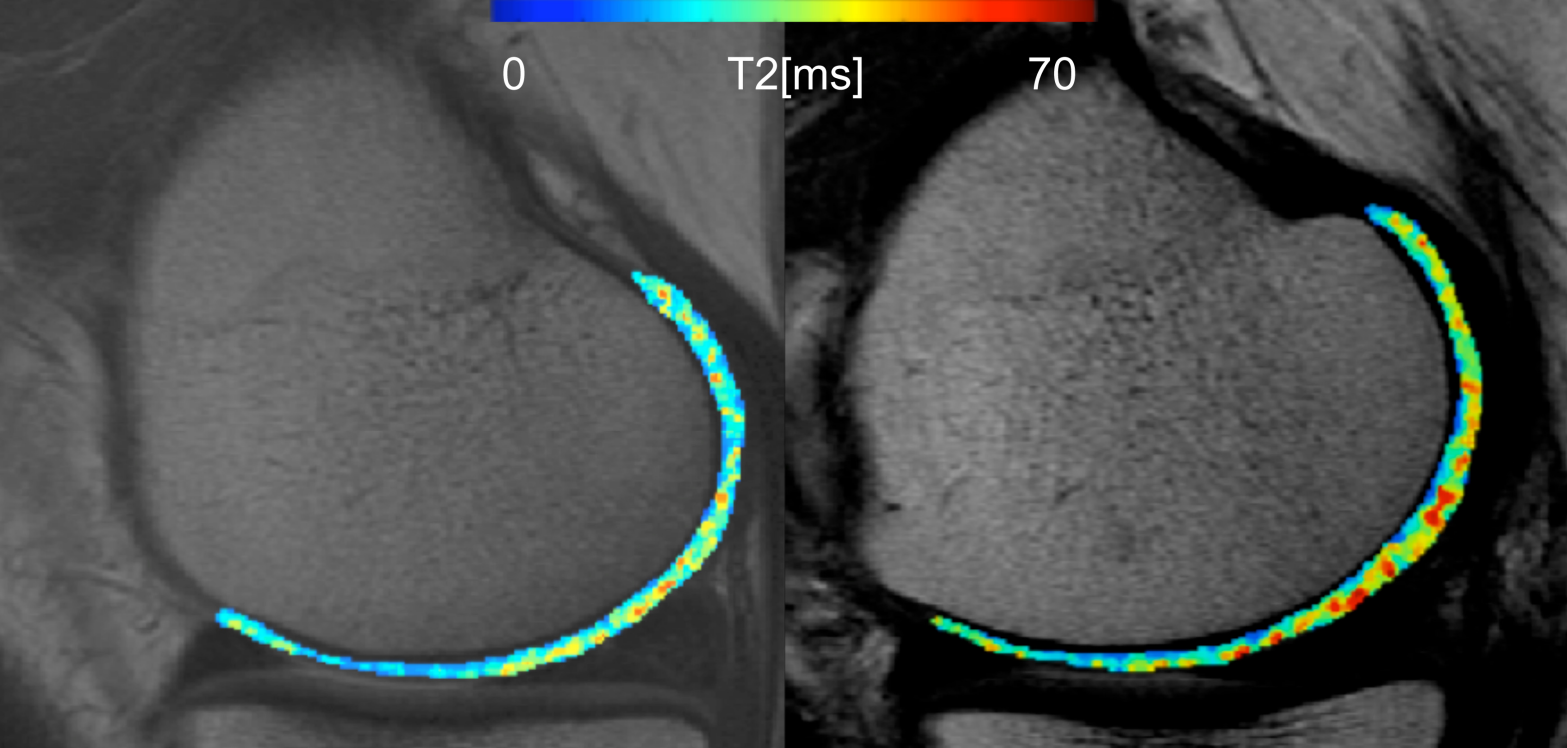
**Representative T**_**2 **_**maps from a subject from the control cohort (left) and a subject from the incidence cohort (right)**. Cartilage T_2 _maps are median-filtered with a 3 × 3 kernal for visualization. Both subjects have no cartilage abnormalities (cartilage whole-organ magnetic resonance imaging scores (WORMS) = 0) and no pain (Western Ontario and McMaster Universities Osteoarthritis Index pain = 0); however, the subject from the incidence cohort has elevated mean T_2 _(39.12 versus 33.39 ms), elevated grey level co-occurrence matrix (GLCM) variance (311.63 versus 190.50), elevated GLCM contrast (466.16 versus 266.82), and elevated GLCM entropy (7.17 versus 6.80). Also, the control subject has intrasubstance abnormalities in the medial posterior meniscus (meniscus WORMS = 1). All other meniscus regions had no abnormalities (meniscus WORMS = 0).

The global mean T_2_, GLCM contrast, and GLCM variance (medial femur, medial tibia, and average of all compartments) were significantly (*P *< 0.05) elevated in the incidence group compared with the control group. Mean T_2 _in the medial femur was greater in the incidence cohort (37.68 ± 2.28 ms) than in the control cohort (36.85 ± 2.16 ms). GLCM entropy (medial femur, medial tibia, and average of all compartments) was elevated in the incidence group but the differences were not significant (*P *> 0.05). Table 7 summarizes the average values of T_2 _parameters in the incidence and control groups. An additional exploratory analysis of remaining compartments demonstrated similar results, but they were not significant. Subjects at risk for OA had elevated mean T_2_, GLCM contrast, and GLCM variance in the lateral femur (*P *> 0.05), the lateral tibia (*P *> 0.05), and the patella (*P *> 0.05). The incidence and control groups had a 0.21 SD difference in mean T_2_, a 0.28 SD difference in entropy, a 0.31 SD difference in variance, and a 0.14 SD difference in entropy (average of all compartments). Figure 1 illustrates two representative T_2 _maps from a control and a subject at risk for OA, respectively. While both subjects do not have cartilage abnormalities (WORMS = 0), the subject from the incidence cohort has greater mean T_2_, GLCM contrast, GLCM variance, and GLCM entropy of cartilage T_2_.

**Table 7:** Average values of T_2 _parameters in the incidence and control groups

Parameter		**Compartment**^**a**^	Incidence group (n = 92)	Control group (n = 53)	*P *value unadjusted	***P *value adjusted**^**b**^	**Coefficient**^**c**^	95% confidence interval	
T_2_	Mean, ms	All	32.65 ± 1.55	32.07 ± 1.38	0.056	**0.018**	-0.72	-1.31	-0.12
		MF	37.68 ± 2.28	36.85 ± 2.16	0.042	**0.003**	-1.18	-1.94	-0.42
		MT	30.27 ± 1.88	29.51 ± 1.77	0.029	**0.036**	-0.72	-1.39	-0.04
GLCM	Contrast	All	248.67 ± 38.39	227.41 ± 35.00	0.005	**0.003**	-22.81	-37.48	-8.13
		MF	364.17 ± 68.98	333.37 ± 66.58	0.013	**0.009**	-32.97	-57.72	-8.21
		MT	257.30 ± 63.19	226.96 ± 47.83	0.004	**0.013**	-25.40	-45.49	-5.48
	Entropy	All	6.34 ± 0.16	6.30 ± 0.19	0.142	0.286	-0.03	-0.10	0.03
		MF	6.96 ± 0.17	6.90 ± 0.17	0.055	0.062	-0.06	-0.12	0.003
		MT	6.17 ± 0.28	6.09 ± 0.34	0.050	0.127	-0.07	-0.15	0.02
	Variance	All	187.77 ± 26.69	171.74 ± 24.57	0.003	**0.002**	-16.60	-27.09	-6.27
		MF	255.64 ± 42.38	233.10 ± 38.60	0.003	**0.001**	-24.86	-39.82	-9.91
		MT	183.65 ± 37.88	162.66 ± 32.01	0.001	**0.005**	-17.89	-30.31	-5.47

^a^All, average of all compartments: medial femur (MF), medial tibia (MT), lateral femur, lateral tibia, and patella. ^b^*P *value adjusted for age, gender, and body mass index (BMI). ^c^The coefficient is the difference in the T_2 _parameter between the incidence and control groups, adjusted for age, gender, and BMI. A coefficient below 0 indicates that the control group has a lower estimated T_2 _parameter in comparison with the incidence group. GLCM, grey level co-occurrence matrix.

**Figure 2:**
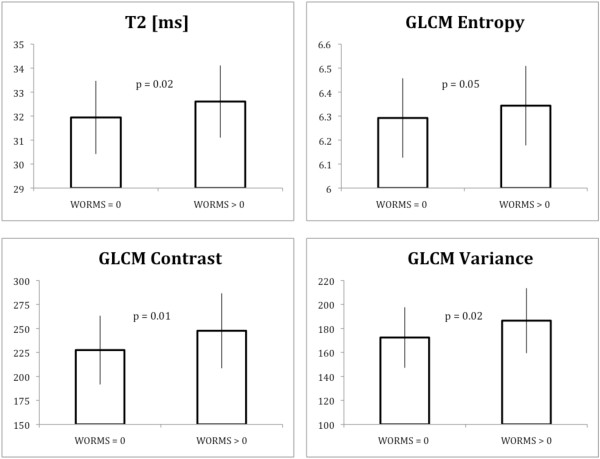
**Comparison of T**_**2 **_**and texture parameters in subjects with cartilage abnormalities and those without them**. Subjects with cartilage abnormalities (cartilage whole-organ magnetic resonance imaging scores (WORMS) of greater than 0, n = 92) have elevated mean T_2_, grey level co-occurrence matrix (GLCM) entropy, GLCM contrast, and GLCM variance in comparison with subjects without abnormalities (cartilage WORMS = 0, n = 41). Values are averaged among all compartments.

Subjects with cartilage abnormalities (cartilage WORMS of greater than 0: n = 92) had significantly (*P *< 0.05) elevated cartilage T_2 _parameters (mean T_2_, GLCM variance, GLCM contrast, and GLCM entropy) than subjects without abnormalities (WORMS = 0: n = 41) in the average of all compartments, in the medial femur, and the patella. The remaining compartments did not demonstrate a significant relationship (*P *> 0.05). This analysis pooled the incidence and control cohorts and accounted for group in the regression model. Similar trends were observed when subdividing the analysis by group. Note that eight subjects from the control group and four subjects from the incidence group did not have WORMS readings available. Figure 2 illustrates that the mean T_2_, GLCM contrast, GLCM variance, and GLCM entropy are significantly elevated in subjects with cartilage abnormalities.

A positive relationship between cartilage WORMS max score and T_2 _parameters (mean cartilage T_2 _(partial correlation adjusting for age, gender, and BMI) *r *= 0.31, *P *= 0.0007), GLCM variance (*r *= 0.18, *P *= 0.04), GLCM contrast (*r *= 0.17, *P *= 0.03), and GLCM entropy (*r *= 0.31, *P *= 0.09) was demonstrated in the medial femur and across both the control and incidence groups. The remaining compartments demonstrated similar trends but the correlations were not significant (*P *> 0.05).

## Discussion

This study evaluated the differences in knee morphology and biochemical composition in the incidence and control groups of the OAI. While there was no significant difference in the prevalence of knee abnormalities (cartilage lesions, bone marrow lesions, and meniscus lesions) between the incidence and control groups, T_2 _parameters (mean T_2_, GLCM contrast, and GLCM variance) were significantly elevated in the incidence group. These results demonstrate that subjects at risk for OA may experience early breakdown of the cartilage extracellular matrix (ECM), such as changes to the collagen structure and increased mobility of water, prior to cartilage degeneration. It is interesting that both subject groups had neither pain (WOMAC pain = 0) nor radiographic evidence (KL score of 0 in the tibiofemoral joint) of OA at baseline yet had varying biochemical compositions. These results suggest that T_2 _mapping may be useful in detecting early arthritic biochemical cartilage changes that precede morphologic degeneration in OA.

While radiography did not demonstrate joint space narrowing or osteophytes in either subject group, MRI detected cartilage and meniscus defects in both groups. The patellar cartilage had the highest prevalence of abnormalities compared with the other compartments, and this corroborates previous studies in athletes [35], candidates for cartilage repair surgery [36], and controls and subjects who developed frequent knee symptoms over 15 months [34]. The posterior horn of the medial meniscus had the highest prevalence of meniscus degeneration, and this has been previously reported [37-39]. Previous studies have demonstrated discordant findings between radiographic and arthroscopic joint damage: subjects with normal radiographic KL scores often demonstrated advanced OA when arthroscopy was used [14]. Thus, soft tissue degeneration in the knee may not closely correspond with joint space narrowing [14,15], and radiography may not be optimal for assessing early-stage arthritic joint degeneration.

Interestingly, the prevalence of cartilage and meniscus morphologic abnormalities was similar between controls and subjects at risk for OA. One might expect that subjects at risk for OA may have an increased number of morphologic abnormalities; however, this was not the case in this study. Similar results were reported in a study by Crema and colleagues [39], who demonstrated that the prevalence of meniscal abnormalities was similar between patients with OA (frequent knee symptoms and KL score of 2 to 3) and controls. In addition, Javaid and colleagues [34] reported that the prevalence of cartilage lesions (any feature damage, whole knee) was similar between OA subjects (KL score of 0 at baseline) who developed frequent knee symptoms over 15 months (80.6%) and controls (67.2%); however, severe cartilage lesions were significantly more prevalent in subjects with OA (22.2% in subjects with OA and 8.6% in controls). The results of these studies suggest that control subjects have a similar prevalence of morphologic abnormalities as those at risk for OA and those with mild/moderate OA; thus, the use of morphologic grading to discriminate between subjects with early OA and controls may be challenging.

While the prevalence of morphologic abnormalities was similar between groups, the mean T_2 _significantly differed, indicating that subjects at risk for OA have altered cartilage biochemistry. Cartilage T_2 _relaxation time is sensitive to the mobility of water in cartilage tissue [40], water content [41], and collagen fiber orientation [42]; changes to these elements of the ECM characterize the initial stages of early OA, eventually leading to gross joint degeneration as detected by morphologic MRI. The elevation of cartilage T_2 _suggests that early cartilage biochemical changes may be of primary interest when assessing subjects at risk for OA.

While elevated mean T_2 _values are associated with OA, the heterogeneous nature of cartilage tissue is also an important consideration when quantifying cartilage tissue integrity. Nissi and colleagues [43] reported that healthy bovine cartilage samples showed a laminar appearance while spontaneously degenerated bovine cartilage tissue did not, demonstrating changes in the distribution of cartilage ECM components with degeneration. In addition, previous studies have shown varying T_2 _relaxation times from the cartilage-bone interface to the joint surface [24,40,44-47] and varying spatial patterns of T_2 _values in osteoarthritic cartilage [48]. Therefore, quantifying only mean values of cartilage T_2 _may mask important information regarding the spatial changes occurring in the ECM during degeneration.

The results of this study demonstrated that subjects at risk for OA have localized variations in their cartilage composition, as evidenced by their elevated GLCM contrast, GLCM entropy, and GLCM variance. Specifically, GLCM contrast is a measure of the differences in neighboring pixel values; high contrast signifies that many pixels with different values are neighboring. GLCM entropy is a measure of disorder in an image; high entropy signifies that the probability of pixel co-occurrence is uniform throughout an image. GLCM variance is a measure of the distribution of pixels about the mean; high variance signifies a high dispersion of co-occurrences of relaxation times. Previous studies have demonstrated differences in the spatial distribution of cartilage relaxation times in subjects with OA and those without OA. For example, Carballido-Gamio and colleagues [9] demonstrated elevated GLCM contrast and GLCM entropy of T1 relaxation time in rotating frame (T_1ρ_) and T_2 _in subjects with mild OA as compared with controls; Li and colleagues [8] demonstrated elevated GLCM contrast and entropy of patellar cartilage T_1ρ _in patients with OA compared with controls; and Blumenkrantz and colleagues [7] demonstrated elevated GLCM entropy of cartilage T_2 _in patients with OA as compared with controls. Additionally, Burstein and colleagues [49] illustrated a loss of normal spatial dependency of cartilage T_2 _relaxation times in a patient with anterior knee pain and chronic chondral injury. The authors suggested that areas of high T_2_-weighted signal (frequently associated with cartilage injury) are often adjacent to areas with low T_2_. Such degenerative changes in cartilage tissue due to disease or injury are reflected by the spatial distribution of T_2 _values and can be quantified by GLCM texture analysis.

This study demonstrated that cartilage abnormalities were associated with elevated and more heterogeneous cartilage T_2 _values, corroborating previous research: Blumenkrantz and colleagues [50] found an association between cartilage T_2 _and cartilage thickness, Mosher and colleagues [51] reported changes in cartilage T_2 _and cartilage thickness after running, Stahl and colleagues [52] reported associations between cartilage T_2 _and cartilage volume and thickness, and Stehling and colleagues [20] demonstrated a relationship between patellar cartilage T_2 _and cartilage morphology (WORMS). These results highlight the complex interrelationship between biochemical cartilage changes and consequent morphologic cartilage loss and suggest that biochemical cartilage composition as measured by T_2 _may be associated with cartilage loss.

Several limitations are pertinent to this study: it may have been useful to subdivide the cartilage into weight-bearing and non-weight-bearing regions. To minimize errors due to multiple comparisons, this type of segmentation was not performed. Furthermore, other techniques such as dGEMRIC (delayed gadolinium-enhanced MRI of cartilage) or T_1ρ _may have been useful in investigating the ECM during OA progression; however, this study did not employ these methods, as the required MRI sequences were not acquired in the OAI protocol.

Because the feasibility of GLCM texture analysis by using the OAI dataset was demonstrated by Carballido-Gamio and colleagues [53], their study provided the foundation for the present study. The present study evaluated a larger subject cohort (145 versus 13 subjects), examined distinct subject groups (we examined subjects at risk for OA and healthy controls while Carballido-Gamio and colleagues [53] examined subjects with symptomatic and radiographic OA), and assessed joint morphology in addition to cartilage T_2_. Thus, the present study is unique in assessing the spatial distribution of cartilage T_2 _values by using GLCM texture analysis in a large cohort at risk for OA.

## Conclusions

This study demonstrated that subjects at risk for OA have both higher and more heterogeneous T_2 _values than controls and that subjects with cartilage abnormalities have elevated cartilage T_2 _parameters compared with subjects without abnormalities. While joint morphology was similar in both groups, cartilage T_2 _parameters showed significant differences, suggesting that T_2 _relaxation time may be a valuable early marker for OA.

## Abbreviations

2D: two-dimensional; BMI: body mass index; CG: control group; CV: coefficient of variation; ECM: extracellular matrix; FSE: fast spin-echo; GLCM: grey level co-occurrence matrix; ICC: intraclass correlation coefficient; IG: incidence group; IW: intermediate-weighted; KL: Kellgren-Lawrence; MR: magnetic resonance; MRI: magnetic resonance imaging; MSME: multi-slice multi-echo; OA: osteoarthritis; OAI: Osteoarthritis Initiative; RMS: root mean square; SD: standard deviation; T_1ρ_: T1 relaxation time in rotating frame; TE: echo time; WOMAC: Western Ontario and McMaster Universities Osteoarthritis Index; WORMS: whole-organ magnetic resonance imaging score; WORMS max score: the maximum of the whole-organ magnetic resonance imaging scores in all compartments per patient.

## Competing interests

The authors declare that they have no competing interests.

## Authors' contributions

GBJ assisted with the study design, performed T_2 _assessment and statistical analysis, and drafted the manuscript. TB assisted in designing the study, supervised the cartilage segmentation, and helped interpret the data and perform the analysis. JC-G developed the software for T_2 _mapping quantification and texture analysis. LN performed WORMS grading and cartilage segmentations. WV performed WORMS grading. HA performed cartilage segmentation. JAL participated in the study design and patient selection. CEM advised with and helped perform the statistical analysis. SM participated in the conceptual design of the study, data interpretation, and analysis. TML participated in the design of the study, interpretation of data, performing WORMS scoring, and manuscript revision. All authors read and approved the final manuscript.

## References

[B1] Handout on Health: Osteoarthritis, National Institute of Arthritis and Musculoskeletal and Skin Diseases http://www.niams.nih.gov/Health_Info/Osteoarthritis/

[B2] Buckwalter J Mankin H Articular cartilage. Part II: Degeneration and osteoarthrosis, repair, regeneration, and transplantation Am J Sports Med 1997 79 612 632

[B3] Dijkgraaf LC de Bont LG Boering G Liem RS The structure, biochemistry, and metabolism of osteoarthritic cartilage: a review of the literature J Oral Maxillofac Surg 1995 53 1182 1192 10.1016/0278-2391(95)90632-0 7562173

[B4] Mankin HJ The reaction of articular cartilage to injury and osteoarthritis (first of two parts) N Engl J Med 1974 291 1285 1292 10.1056/NEJM197412122912406 4610388

[B5] Mosher TJ Dardzinski BJ Cartilage MRI T2 relaxation time mapping: overview and applications Semin Musculoskelet Radiol 2004 8 355 368 10.1055/s-2004-861764 15643574

[B6] Dunn TC Lu Y Jin H Ries MD Majumdar S T2 relaxation time of cartilage at MR imaging: comparison with severity of knee osteoarthritis Radiology 2004 232 592 598 10.1148/radiol.2322030976 15215540 PMC4447089

[B7] Blumenkrantz G Stahl R Carballido-Gamio J Zhao S Lu Y Munoz T Hellio Le Graverand-Gastineau MP Jain SK Link TM Majumdar S The feasibility of characterizing the spatial distribution of cartilage T(2) using texture analysis Osteoarthritis Cartilage 2008 16 584 590 10.1016/j.joca.2007.10.019 18337129 PMC2838772

[B8] Li X Pai A Blumenkrantz G Carballido-Gamio J Link T Ma B Ries M Majumdar S Spatial distribution and relationship of T1rho and T2 relaxation times in knee cartilage with osteoarthritis Magn Reson Med 2009 61 1310 1318 10.1002/mrm.21877 19319904 PMC2753277

[B9] Carballido-Gamio J Stahl R Gabrielle B Adan R Sharmila M Spatial analysis of magnetic resonance T1rho and T2 relaxation times improves classification between subjects with and without osteoarthritis Med Phys 2009 36 4059 4067 10.1118/1.3187228 19810478 PMC3908744

[B10] Haralick RM Shanmugam K Dinstein I Textural features for image classification IEEE Transactions on Systems, Man, and Cybernetics 1973 SMC-3 610 618

[B11] Link TM Steinbach LS Ghosh S Ries M Lu Y Lane N Majumdar S Osteoarthritis: MR imaging findings in different stages of disease and correlation with clinical findings Radiology 2003 226 373 381 10.1148/radiol.2262012190 12563128

[B12] Englund M Roos E Lohmander L Impact of type of meniscal tear on radiographic and symptomatic knee osteoarthritis: a sixteen year followup of meniscectomy with matched controls Arthritis Rheum 2003 48 2178 2187 10.1002/art.11088 12905471

[B13] Raynauld J Martel-Pelletier J Berthiaume M Beaudoin G Choquette D Haraoui B Tannenbaum H Meyer J Beary J Cline G Long term evaluation of disease progression through the quantitative magnetic resonance imaging of symptomatic knee osteoarthritis patients: correlation with clinical symptoms and radiographic changes Arthritis Res Ther 2006 8 R21 16507119 10.1186/ar1875 PMC1526551

[B14] Brandt K Fife R Braunstein E Katz B Radiographic grading of the severity of knee osteoarthritis: relation of the Kellgren and Lawrence grade to a grade based on joint space narrowing, and correlation with arthroscopic evidence of articular cartilage degeneration Arthritis Rheum 1991 34 1381 1386 1953815 10.1002/art.1780341106

[B15] Lysholm J Hamberg P Gillquist J The correlation between osteoarthrosis as seen on radiographs and on arthroscopy Arthroscopy 1987 3 161 10.1016/S0749-8063(87)80058-0 3675786

[B16] Peterfy CG Guermazi A Zaim S Tirman PF Miaux Y White D Kothari M Lu Y Fye K Zhao S Genant HK Whole-Organ Magnetic Resonance Imaging Score (WORMS) of the knee in osteoarthritis Osteoarthritis Cartilage 2004 12 177 190 10.1016/j.joca.2003.11.003 14972335

[B17] Nevitt MC Felson DT Lester G The Osteoarthritis Initiative: Protocol for the Cohort Study UC San Francisco; Boston University; National Institute of Arthritis, Musculoskeletal and Skin Diseases http://oai.epi-ucsf.org/datarelease/docs/StudyDesignProtocol.pdf

[B18] Kellgren J Lawrence J Radiologic assessment of osteoarthritis Ann Rheum Dis 1957 16 494 502 10.1136/ard.16.4.494 13498604 PMC1006995

[B19] Peterfy C Schneider E Nevitt M The osteoarthritis initiative: report on the design rationale for the magnetic resonance imaging protocol for the knee Osteoarthritis Cartilage 2008 16 1433 10.1016/j.joca.2008.06.016 18786841 PMC3048821

[B20] Stehling C Liebl H Krug R Lane NE Nevitt MC Lynch J McCulloch CE Link TM Patellar cartilage: T2 values and morphologic abnormalities at 3.0-T MR imaging in relation to physical activity in asymptomatic subjects from the osteoarthritis initiative Radiology 2010 254 509 520 10.1148/radiol.09090596 20019141 PMC2809928

[B21] Pan J Stehling C Muller-Hocker C Schwaiger BJ Lynch J McCulloch CE Nevitt MC Link TM Vastus lateralis/vastus medialis cross-sectional area ratio impacts presence and degree of knee joint abnormalities and cartilage T2 determined with 3T MRI - an analysis from the incidence cohort of the Osteoarthritis Initiative Osteoarthritis Cartilage 2011 19 65 73 10.1016/j.joca.2010.10.023 21044692 PMC3027210

[B22] Miller AJ Joseph PM The use of power images to perform quantitative analysis on low SNR MR images Magn Reson Imaging 1993 11 1051 1056 10.1016/0730-725X(93)90225-3 8231670

[B23] Raya J Dietrich O Horng A Weber J Reiser M Glaser C T2 measurement in articular cartilage: impact of the fitting method on accuracy and precision at low SNR Magn Reson Med 2010 63 181 193 19859960 10.1002/mrm.22178

[B24] Smith HE Mosher TJ Dardzinski BJ Collins BG Collins CM Yang QX Schmithorst VJ Smith MB Spatial variation in cartilage T2 of the knee J Magn Reson Imaging 2001 14 50 55 10.1002/jmri.1150 11436214

[B25] Maier CF Tan SG Hariharan H Potter HG T2 quantitation of articular cartilage at 1.5 T J Magn Reson Imaging 2003 17 358 364 10.1002/jmri.10263 12594727

[B26] Peterfy CG Gold G Eckstein F Cicuttini F Dardzinski B Stevens R MRI protocols for whole-organ assessment of the knee in osteoarthritis Osteoarthritis Cartilage 2006 14 Suppl A A95 111 16750915 10.1016/j.joca.2006.02.029

[B27] Stahl R Luke A Ma CB Krug R Steinbach L Majumdar S Link TM Prevalence of pathologic findings in asymptomatic knees of marathon runners before and after a competition in comparison with physically active subjects-a 3.0 T magnetic resonance imaging study Skeletal Radiol 2008 37 627 638 10.1007/s00256-008-0491-y 18463868

[B28] Shrout PE Fleiss JL Intraclass correlations: uses in assessing rater reliability Psychol Bull 1979 86 420 428 18839484 10.1037//0033-2909.86.2.420

[B29] Norman GR Streiner DL Biostatistics: The Bare Essentials 2008 Beijing, China: People's Medical Publishing House

[B30] Fleiss JL Cohen J The equivalence of weighted kappa and the intraclass correlation coefficient as measures of reliability Educational and Psychological Measurement 1973 33 613 619 10.1177/001316447303300309

[B31] Glüer CC Blake G Blunt BA Jergas M Genant HK Accurate assessment of precision errors: how to measure the reproducibility of bone densitometry techniques Osteoporosis Int 1995 5 262 270 10.1007/BF01774016 7492865

[B32] Matthews BF Composition of articular cartilage in osteoarthritis Br Med J 1953 2 660 10.1136/bmj.2.4837.660 13082071 PMC2029523

[B33] Bonnin M Osteoarthritis of the Knee 2008 New York: Springer

[B34] Javaid M Lynch J Tolstykh I Guermazi A Roemer F Aliabadi P McCulloch C Curtis J Felson D Lane N Pre-radiographic MRI findings are associated with onset of knee symptoms: the most study Osteoarthritis Cartilage 2010 18 323 328 10.1016/j.joca.2009.11.002 19919856 PMC2990960

[B35] Flanigan DC Harris JD Trinh TQ Siston RA Brophy RH Prevalence of chondral defects in athletes' knees: a systematic review Med Sci Sports Exerc 2010 42 1795 1801 10.1249/MSS.0b013e3181d9eea0 20216470

[B36] Widuchowski W Widuchowski J Trzaska T Articular cartilage defects: study of 25,124 knee arthroscopies Knee 2007 14 177 182 10.1016/j.knee.2007.02.001 17428666

[B37] Zarins ZA Bolbos RI Pialat JB Link TM Li X Souza RB Majumdar S Cartilage and meniscus assessment using T1rho and T2 measurements in healthy subjects and patients with osteoarthritis Osteoarthritis Cartilage 2010 18 1408 1416 10.1016/j.joca.2010.07.012 20696262 PMC2975868

[B38] Kornick J Trefelner E McCarthy S Lange R Lynch K Jokl P Meniscal abnormalities in the asymptomatic population at MR imaging Radiology 1990 177 463 2217786 10.1148/radiology.177.2.2217786

[B39] Crema MD Guermazi A Li L Nogueira-Barbosa MH Marra MD Roemer FW Eckstein F Hellio Le Graverand MP Wyman BT Hunter DJ The association of prevalent medial meniscal pathology with cartilage loss in the medial tibiofemoral compartment over a 2-year period Osteoarthritis Cartilage 2009 18 336 343 19914195 10.1016/j.joca.2009.11.003

[B40] Mosher TJ Liu Y Yang QX Yao J Smith R Dardzinski BJ Smith MB Age dependency of cartilage magnetic resonance imaging T2 relaxation times in asymptomatic women Arthritis Rheum 2004 50 2820 2828 10.1002/art.20473 15457450

[B41] Liess C Lusse S Karger N Heller M Gluer CC Detection of changes in cartilage water content using MRI T2-mapping *in vivo* Osteoarthritis Cartilage 2002 10 907 913 10.1053/joca.2002.0847 12464550

[B42] Xia Y Magic-angle effect in magnetic resonance imaging of articular cartilage: a review Invest Radiol 2000 35 602 621 10.1097/00004424-200010000-00007 11041155

[B43] Nissi MJ Toyras J Laasanen MS Rieppo J Saarakkala S Lappalainen R Jurvelin JS Nieminen MT Proteoglycan and collagen sensitive MRI evaluation of normal and degenerated articular cartilage J Orthop Res 2004 22 557 564 10.1016/j.orthres.2003.09.008 15099635

[B44] Mosher TJ Smith HE Collins C Liu Y Hancy J Dardzinski BJ Smith MB Change in knee cartilage T2 at MR imaging after running: a feasibility study Radiology 2005 234 245 249 10.1148/radiol.2341040041 15550376

[B45] Mosher TJ Collins CM Smith HE Moser LE Sivarajah RT Dardzinski BJ Smith MB Effect of gender on *in vivo *cartilage magnetic resonance imaging T2 mapping J Magn Reson Imaging 2004 19 323 328 10.1002/jmri.20013 14994301

[B46] White LM Sussman MS Hurtig M Probyn L Tomlinson G Kandel R Cartilage T2 assessment: differentiation of normal hyaline cartilage and reparative tissue after arthroscopic cartilage repair in equine subjects Radiology 2006 241 407 10.1148/radiol.2412051750 17057068

[B47] Hannila I Susanna Raina S Tervonen O Ojala R Nieminen MT Topographical variation of T2 relaxation time in the young adult knee cartilage at 1.5 T Osteoarthritis Cartilage 2009 17 1570 1575 10.1016/j.joca.2009.05.011 19501682

[B48] Dray N Williams A Prasad PV Sharma L Burstein D T2 in an OA population: metrics for reporting data? International Society of Magnetic Resonance in Medicine 2005 Miami, FL 1995

[B49] Burstein D Gray M Mosher T Dardzinski B Measures of molecular composition and structure in osteoarthritis Radiol Clin North Am 2009 47 675 686 10.1016/j.rcl.2009.04.003 19631075

[B50] Blumenkrantz G Lindsey CT Dunn TC Jin H Ries MD Link TM Steinbach LS Majumdar S A pilot, two-year longitudinal study of the interrelationship between trabecular bone and articular cartilage in the osteoarthritic knee Osteoarthritis Cartilage 2004 12 997 1005 10.1016/j.joca.2004.09.001 15564067

[B51] Mosher TJ Liu Y Torok CM Functional cartilage MRI T2 mapping: evaluating the effect of age and training on knee cartilage response to running Osteoarthritis Cartilage 2009 18 358 364 19948266 10.1016/j.joca.2009.11.011 PMC2826588

[B52] Stahl R Blumenkrantz G Carballido-Gamio J Zhao S Munoz T Hellio Le Graverand-Gastineau MP Li X Majumdar S Link TM MRI-derived T2 relaxation times and cartilage morphometry of the tibio-femoral joint in subjects with and without osteoarthritis during a 1-year follow-up Osteoarthritis Cartilage 2007 15 1225 1234 10.1016/j.joca.2007.04.018 17561417

[B53] Carballido-Gamio J Joseph GB Lynch JA Link TM Majumdar S Longitudinal analysis of MRI T2 knee cartilage laminar organization in a subset of patients from the osteoarthritis initiative: a texture approach Magn Reson Med 2010 65 1184 1194 21413082 10.1002/mrm.22693

